# Association of birth weight with corneal power in early adolescence: Results from the National Health and Nutrition Examination Survey (NHANES) 1999–2008

**DOI:** 10.1371/journal.pone.0186723

**Published:** 2017-10-26

**Authors:** Achim Fieß, Alexander K. Schuster, Norbert Pfeiffer, Stefan Nickels

**Affiliations:** Department of Ophthalmology, University Medical Center Mainz, Mainz, Germany; Xiamen University, CHINA

## Abstract

**Purpose:**

To analyze the effect of birth weight on ocular morphology, refraction and visual function in early adolescents aged 12–15 years.

**Material and methods:**

We conducted a secondary data analysis using the public use files from the National Health and Nutrition Examination Survey of the period from 1999 to 2008. Study participants aged 12 to 15 years were included with data on birth weight and ophthalmic parameters including presenting distance visual acuity, objective refraction and keratometry. Visual acuity, sphere, astigmatism in power vectors J_0_ and J_45_, corneal power and corneal astigmatism were evaluated for an association with birth weight. Linear and logistic regression with adjustment for age, sex, ethnicity, survey cycle and birth weight as independent variable were calculated.

**Results:**

Linear regression analysis revealed an association between corneal power and birth weight (per 100g: beta = -0.04, p<0.001) in the univariate analysis, and in the model adjusted for age, sex, ethnicity and NHANES survey cycle (per 100g: beta = -0.04, p<0.001). A lower birth weight was associated with higher corneal power. We found no evidence for an association of visual acuity, sphere, spherical equivalent, J_0_-vector and J_45_-vector of astigmatism, corneal J_0_- or corneal J_45_-vector with birth weight.

**Conclusion:**

Our data demonstrate that low BW is linked to alterations in keratometric power even in early adolescents aged 12–15 years whereas visual acuity and refractive error showed no association.

## Introduction

In recent years, newborns with low birth weight have increased chances to survive. Especially low birth weight is an important parameter indicating prenatal growth restriction. Furthermore, it is assumed that growth restriction during organ development can affect organ morphology and functioning including ocular development and morphology in childhood. For former preterm and/or very low birth weight infants have an increased risk described for reduced visual acuity,[1] higher refractive error,[2] higher astigmatisms,[3] steeper corneal radius,[4] shallower anterior chamber, thicker lens,[5] shorter axial length [4, 6] and altered retinal morphology [7, 8].

Nevertheless, existing data mainly relies on examinations in early infancy. It is less clear whether altered ocular morphology in low birth weight infants lasts until adolescents or even until adulthood. Some authors hypothesized that differences in ocular morphology diminish between former preterm low birth weight infants and former full term neonates in the first seven years of life.[4]

In addition, former analyses were mainly based on case-control studies or hospital-based studies including infants with extremely low birth weight and controls. It is unclear whether these findings can be generalized to the general population. Consequently, it is of clinical importance to clarify and quantify the influence of this maturity parameter on ocular outcome in a population-based investigation to targeted clinical follow up beyond infancy.

Therefore, the purpose of this investigation was to analyze the long-term effects of low birth weight on visual acuity and ocular morphology in early teenage (12–15 years). Our hypothesis was that we would confirm previous findings in a population-based setup, namely an association between low birth weight and lower visual acuity, higher refractive error, higher astigmatism and higher corneal power even in early teenage.

## Materials and methods

The National Health and Nutrition Examination Survey (NHANES) is a representative survey research program to assess the health and nutritional status of adults and children in the United States of America (https://www.cdc.gov/nchs/nhanes/index.htm, last accessed 2017-05-10). Since 1999, data is collected continuously in two-year periods. Approximately 5000 persons from 15 areas are examined annually. Data is collected via questionnaire-based personal interviews at the participant’s home and a subsequent visit of a mobile examination center (MEC). From 1999 to 2008, NHANES included questions about birth weight as well as an ophthalmic examination. Our analyses are based on the NHANES public use files of these survey cycles (https://wwwn.cdc.gov/nchs/nhanes/continuousnhanes/default.aspx, last accessed 2017-05-10).

### Birth weight

Birth weight was collected via the NHANES Early Childhood Questionnaire (ECQ) which surveyed participants up to 15 years of age and asked their responsible adult for health-related data including birth weight in pounds and ounces.

### Ophthalmic data

During the subsequent visit at the mobile examination center, participants aged 12 years or older were asked to participate in an examination of visual function. Presenting distance visual acuity, objective refraction and corneal radius (flat meridian, steep meridian, axis of meridians) were examined using an autorefractor/keratometer (Nidek ARK-760A, Nidek Co. Ltd., Tokyo, Japan) in non-cycloplegic state and taking the average of three measurements. Corneal radius was converted into corneal power using the keratometer index of r = 1.3375. Presenting distance visual acuity was tested in both eyes. This examination was conducted with participants’s own spectacle correction, if available. Objective refraction (sphere, cylinder, axis) and keratometry data (corneal power averaged across the two meridians, difference in corneal power between meridians, axis of the steepest meridian) were obtained in a non-cycloplegic state by taking the average of three measurements.

To be able to analyze the association between birth weight and ophthalmic parameters, we included study participants aged 12 to 15 years who had had both, data on birth weight and an ophthalmic examination.

### Demographic data

Age and sex were reported by parents, as was ethnicity. Ethnicity was provided in the categories Mexican American, other Hispanic, Non-Hispanic white, Non-Hispanic black, and other.

### Exclusion criteria

Exclusion criteria of the analysis were missing proxy-reported birth weight information or a lack of data of the ophthalmic examination. Furthermore, we excluded all negative measurements of corneal power and extreme values of corneal power above 10 diopters.

### Statistical analysis

We calculated birth weight in the metric system (grams) from the reported weight at birth in pounds and ounces and categorized the participants in born with low birth weight (<2500 g), high birth weight (>4100 g), and normal birth weight, as specified in the questionnaire. We only included right eyes in our analysis. Spherical equivalent (SE) was calculated as sphere value plus half the cylindrical power. For visual acuity, we transformed the Snellen equivalent to LogMAR.[9] The category “20/200+” was set to 1.1 LogMAR. All variables were checked for outliers.

Following the approach of Thibos,[10] who applied Fourier analysis to characterize astigmatism components, we calculated the vectors J_0_ and J_45_ for both refractive and corneal astigmatism as follows: J_0_ = —C/2 * cos (2α) and J_45_ = —C/2 * sin (2α); α is the cylindrical axis, and C is the cylinder power. J_0_ represents the power vector matching the cylinder power of the vertical (90°) and horizontal (180°) meridians. Positive values correspond to with-the-rule astigmatism, negative values correspond to against-the-rule astigmatism. J_45_ is the power vector corresponding to the cylinder power of the oblique meridians (45° and 135°).

We used mean and standard deviation to describe the distribution of continuous variables which showed an approximately normal distribution. Absolute and relative frequencies were used to describe categorical variables.

For continuous variables of interest, we used weighted linear regression models, and for categorical data we conducted weighted logistic regression analyses.

As univariate analysis, one model was built for each of the following outcome variable (visual acuity, sphere, spherical equivalent, J_0_-vector and J_45_-vector of astigmatism, corneal power, corneal J_0_- and corneal J_45_-vector) with birth weight as independent variable. Multivariable regression models included age, sex, ethnicity and NHANES survey cycle. For visual acuity, we additionally adjusted for wearing distance glasses or contact lenses.

To account for the complex survey design, we followed the advice of the NHANES analytical guidelines and used combined sample weights for the analyses.[11] The variance estimation used Taylor Series Linearization based on the primary sampling units and strata. We repeated the analysis of refraction outcomes restricted to myopic participants (defined by SE < -0.5 diopters). Furthermore, we repeated the analyses based on categorized birth weight with an additional category for very low birth weight (<1500 g). We used R version 3.3.0 with Rstudio version 1.0.136 and the packages nhanesA (version 0.6.4.3.3), ggplot2 (version 2.1.0), survey (version 3.31–5), knitr (version 1.15.1), and table one (version 0.7.3). P-values should be regarded as a continuous measure of evidence and should be interpreted with care, given the exploratory character of this analysis. To allow for replication of our analyses, we provide the source code on github (https://github.com/snickels/nhanes_bw_vf).

## Results

### Sample description

We included 4801 NHANES participants of the survey cycles 1999–2008 with available information on birth weight and an ophthalmic examination. For corneal power we excluded negative values (n = 48) and extreme values above 10 diopters (n = 5), as they are highly suspected to be measurement or data entry errors.

Age at examination of the study sample was 13.98 +/- 1.16 years and 51% were female. 34% were Mexican Americans, 5% other Hispanics, 27% non-Hispanic Whites and 31% non-Hispanics Blacks, 4% had another ethnicity. The mean birth weight was 3298 +/- 654 g, 461 (10%) persons had a birth weight below 2500g and 400 (8%) persons had a birth weight over 4100g. Data of general and ocular parameters with stratification on birth weight are reported in Table 1.

**Table 1:** Characteristics of NHANES 1999–2008 sample with both birth weight and ophthalmic data available.

	Normal birth weight (2500 g—4100 g)	Low birth weight (<2500 g)	High birth weight (>4100 g)
n	3940	461	400
Female sex	2036 (51.7%)	247 (53.6%)	150 (37.5%)
Age at examination [years]	13.98 (1.16)	13.97 (1.18)	14.00 (1.12)
Mexican American ethnicity	1317 (33.4%)	128 (27.8%)	161 (40.2%)
Other Hispanic ethnicity	199 (5.1%)	23 (5.0%)	16 (4.0%)
Non-Hispanic white ethnicity	1072 (27.2%)	83 (18.0%)	116 (29.0%)
Non-Hispanic black ethnicity	1184 (30.1%)	209 (45.3%)	94 (23.5%)
Other ethnicity	168 (4.3%)	18 (3.9%)	13 (3.2%)
**Ocular parameters (right eyes):**			
Visual acuity [LogMAR]	0.16 (0.24)	0.17 (0.23)	0.16 (0.24)
Distance glasses	1141 (29.0%)	118 (25.6%)	115 (28.7%)
Sphere [dioptres]	-1.04 (1.81)	-0.90 (1.90)	-1.18 (1.97)
Cylinder [dioptres]	0.61 (0.68)	0.65 (0.74)	0.62 (0.70)
J_0_-vector of astigmatism	0.04 (0.41)	0.05 (0.42)	0.08 (0.42)
J_45_-vector of astigmatism	-0.01 (0.19)	-0.01 (0.25)	0.01 (0.19)
Spherical equivalent [dioptres]	-0.73 (1.70)	-0.57 (1.81)	-0.88 (1.86)
Corneal power, average [dioptres]	43.45 (1.53)	43.83 (1.72)	43.05 (1.61)
Corneal Cylinder [dioptres]	1.02 (1.11)	1.02 (0.74)	1.00 (0.68)
Corneal J_0_-vector	0.40 (0.52)	0.41 (0.38)	0.43 (0.37)
Corneal J_45_-vector	0.04 (0.37)	0.05 (0.29)	0.04 (0.21)

### Regression results

**Fig 1:**
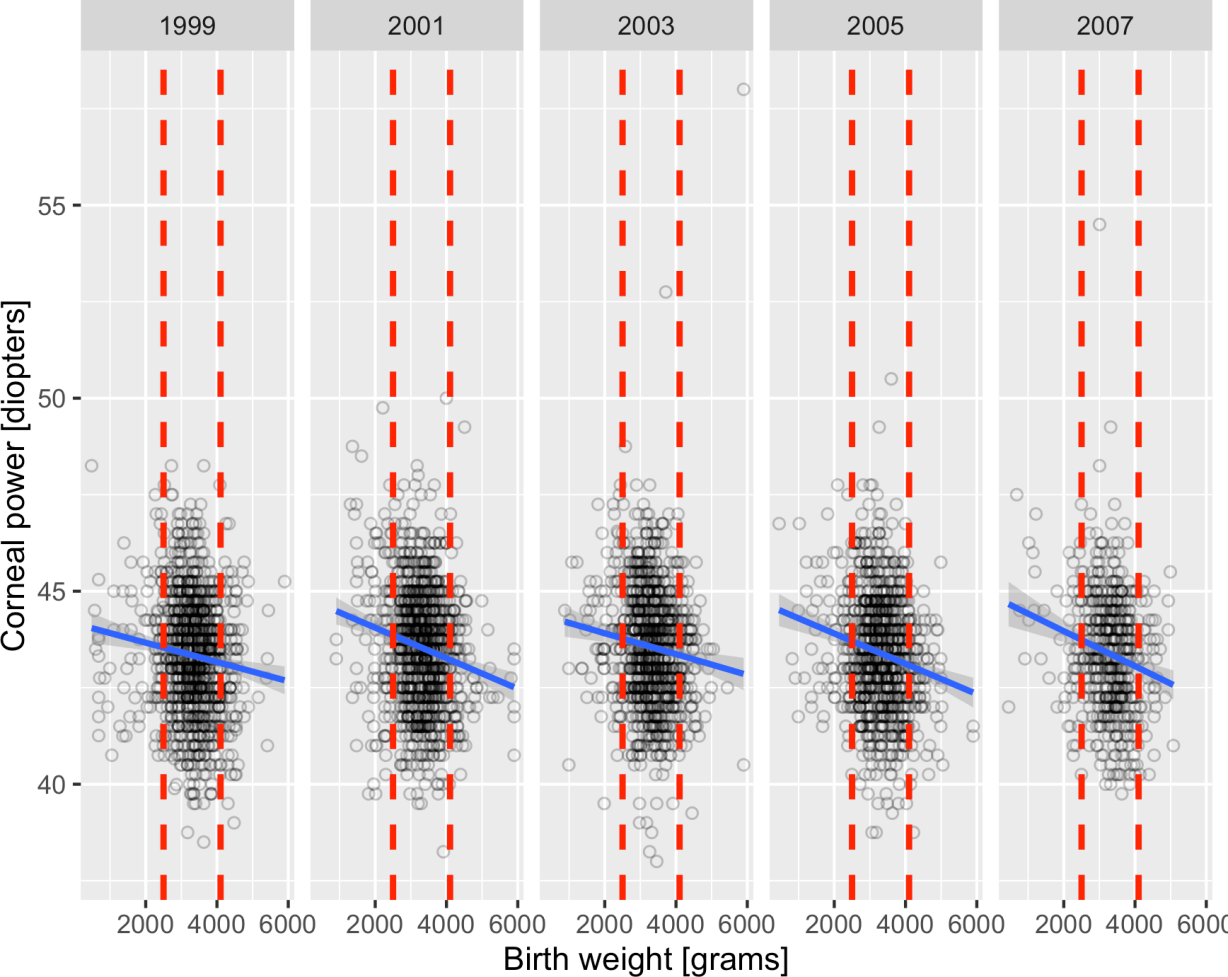
The association of corneal power (right eyes) with birth weight in the NHANES 1999–2008. Legend: Split by survey cycles. Red dashed lines represent the birth weight category boundaries (2500g, 4100g) used in the categorical regression. Blue lines represent regression lines with grey 95% confidence limits.

Linear regression analysis revealed an association between corneal power and birth weight (per 100g: beta = -0.04, p<0.001) in the univariate analysis (Fig 1), and in the model adjusted for age, sex, ethnicity and NHANES examination cycle (per 100g: beta = -0.04, p<0.001). A lower birth weight was associated to higher corneal power. We repeated the analysis restricted to the normal birth weight group (per 100g: beta = -0.04, p<0.001, both crude and adjusted models). Visual acuity, sphere, spherical equivalent, J_0_-vector and J_45_-vector of astigmatism, corneal J_0_- and corneal J_45_-vector were not associated to birth weight in the respective models (Table 2).

**Table 2:** The association of birth weight (continuous) with visual acuity, refraction and keratometry in separate models in the NHANES 1999–2008.

	Crude analysis		Adjusted model*	
	Estimate per 100g [95% confidence interval]	P value	Estimate per 100g [95% confidence interval]	P value
Visual acuity [LogMAR] **	0 [0; 0]	0.17	0 [0; 0]	0.90
Sphere [dioptres]	-0.01 [-0.02; 0.01]	0.33	-0.01 [-0.02; 0]	0.16
Spherical equivalent [dioptres]	-0.01 [-0.02; 0.01]	0.32	-0.01 [-0.02; 0]	0.17
J_0_-vector of astigmatisms	0 [0; 0]	0.79	0 [0; 0]	0.26
J_45_-vector of astigmatisms	0 [0; 0]	0.03	0 [0; 0]	0.06
Corneal power, average [dioptres]	-0.04 [-0.05; -0.03]	1.15e-11	-0.04 [-0.05–0.03]	1.31e-11
Corneal J_0_-vector	0 [0; 0]	0.91	0 [0; 0]	0.14
Corneal J_45_-vector	0 [0; 0]	0.61	0 [0; 0]	0.43

*Results from the multivariable linear regression models adjusted for age, sex, ethnicity, survey cycle, and with consideration of the study sample structure.

**additional adjustment for distance correction

**Fig 2:**
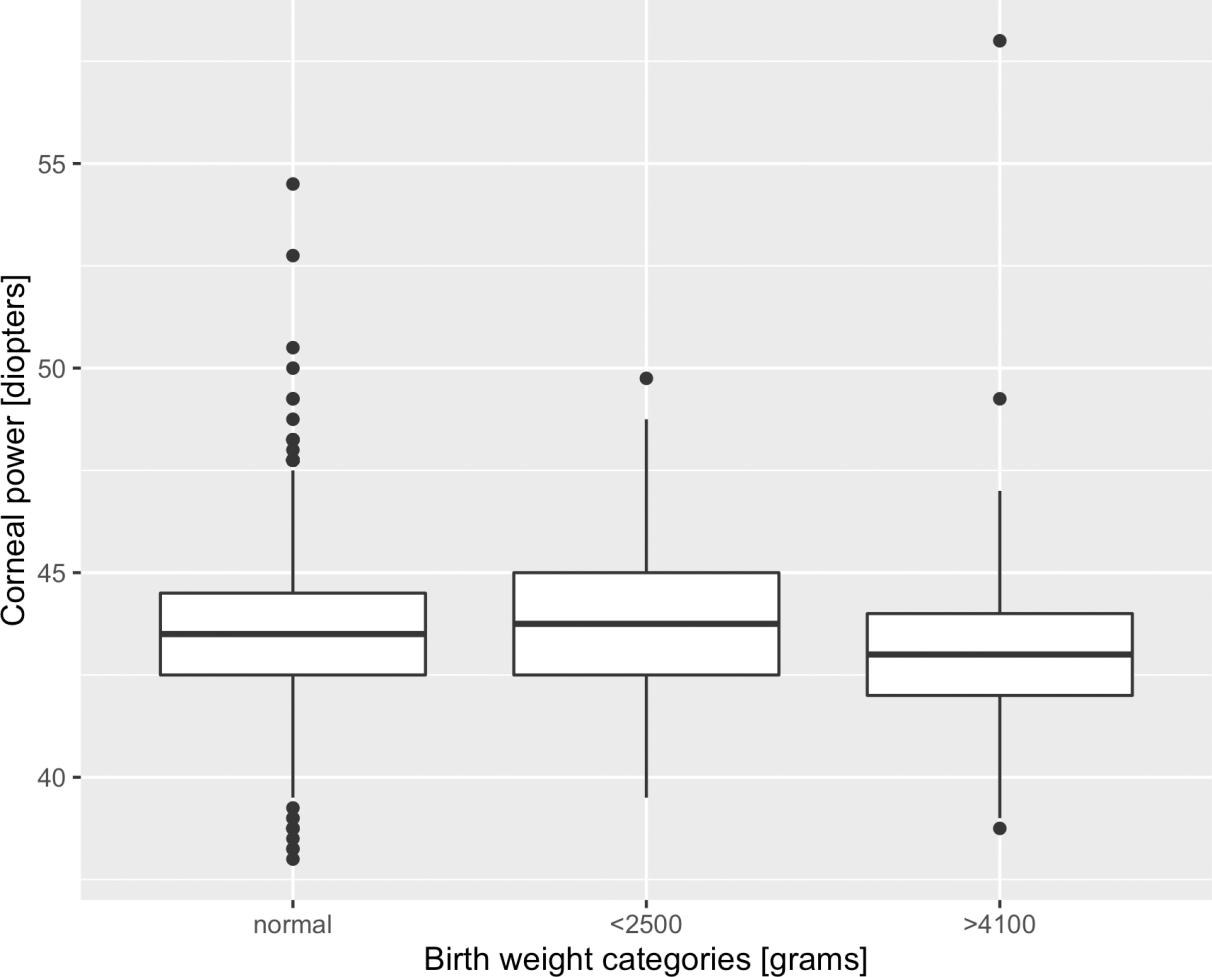
Corneal power (right eyes) by birth weight categories in the NHANES 1999–2008.

With respect to birth weight groups, a 0.70 diopters higher corneal power was associated to birth weight below 2500g compared to normal birth weight (2500-4100g) in the multivariable linear regression model (p<0.001; Fig 2). A birth weight above 4100g was associated a -0.24 diopters lower corneal power compared to normal birth weight (Table 3). No other outcome parameter was associated to low birth weight, while corneal J_0_-vector showed an association to high birth weight in the multivariable regression model.

**Table 3:** The association of self-reported birth weight (categorical) with visual acuity, refraction and keratometry in the NHANES 1999–2008.

	Low birth weight (<2500 g)		High birth weight (>4100 g)	
	Estimate [95% confidence interval]	P value	Estimate [95% confidence interval]	P value
Visual acuity [LogMAR] *	0 [-0.02; 0.03]	0.83	0 [-0.02; 0.03]	0.79
Sphere [dioptres]	0.15 [-0.16; 0.45]	0.35	-0.2 [-0.52; 0.12]	0.23
Spherical equivalent [dioptres]	0.16 [-0.13; 0.46]	0.28	-0.2 [-0.51; 0.11]	0.21
J_0_-vector of astigmatism	0.02 [-0.04; 0.08]	0.43	0.05 [-0.01; 0.11]	0.09
J_45_-vector of astigmatism	-0.01 [-0.03; 0.01]	0.36	0.02 [-0.01; 0.05]	0.17
Corneal power, average [dioptres]	0.70 [0.50; 0.90]	3.62 e-09	-0.24 [-0.44; -0.04]	0.02
Corneal J_0_-vector	0.03 [-0.02; 0.08]	0.25	0.06 [0.01; 0.11]	0.01
Corneal J_45_-vector	0.02 [-0.01; 0.05]	0.28	-0.01 [-0.04; 0.02]	0.45

Legend: Results from the multivariable linear regression models adjusted for age, sex, ethnicity, survey cycle, and with consideration of the study sample structure. Reference was the normal birth weight group (> = 2500 g—< = 4100 g).

*additional adjustment for distance correction

The sensitivity analysis including only myopic subjects revealed similar results. Sphere, spherical equivalent, and J_45_-vector of astigmatism were not linked to birth weight, while J_0_-vector of astigmatism was associated (S1 and S2 Tables).

## Discussion

Our analysis provides data of ocular long term outcome in early adolescents aged 12 to 15 years with respect to their birth weight in a population based setting. It indicates that low birth weight affects corneal configuration even after the first 10 years of life as corneal power is higher in adolescents having had low birth weight. Furthermore, we analysed the impact of birth weight and visual acuity respective refractive error in early adolescents.

### Visual acuity

In contrast to previous reports analyzing children up to late infancy we did not find an association of birth weight with visual acuity. Haugen et al. [12] reported for infants aged 6 to 7 years in a population-based study with former preterm low birth weight infants (gestational age of 22–27 completed weeks or birth weight of 500–999 g) that 46% of participants had subnormal visual acuity of > = 0.1 logMAR. This is in congruence to a study with older children aged up to 10 years. 25% of these study participants with a birth weight ≤ 1500 g had a low visual function, defined as visual acuity > = 0.1 logMAR and/or strabismus and/or subnormal contrast sensitivity.[1] Furthermore, O'Connor et al. [13] compared older children at an age of 10 to 12 years with a birth weight below 1701 g compared to full term children. This study detected a slightly lower visual acuity and contrast sensitivity in children with a low birth weight. In accordance with these results Molloy et al.[14] reported for adolescents (age between 14 and 20 years) with extreme low birth weight (<1000g) or former extreme preterm infants (gestational age < 28 weeks) worse visual acuity, poorer stereopsis and convergence compared to a control group with normal birth weight (>2499g). In contrast to our study, these studies recruited far more study participants with extreme low birth weight, than did our approach investigating this relationship in a population-based study design with only 0.6% (n = 29) subjects with a birth weight below 1000g. Accordingly, our results reflect the continuum of birth weights in a population, while extreme prematurity may show different results.

### Sphere and spherical equivalent

Contrary to previous reports, we found no evidence for an association of low birth weight with increased refractive error. Several reports exist demonstrating a strong association between low birth weight and increased myopic refractive error in early childhood [2, 4, 6, 15, 16]. In a study of extremely preterm infants with GA<27 weeks aged of 2.5 years, about 26% of the children had myopia with less than -3 diopters, hypermetropia greater than +3 diopter, astigmatismus of 2 and more diopter, and/or anisometropia of 2 and more diopter.[16] Overall, for the first decade of life O'Connor et al.[15] observed in a cohort of 293 low birth weight infants (<1701 g) a relatively stable refractive error with a shift of 1 diopter towards myopia. In addition, in a recent study the authors reported that differences of spherical equivalent decreased between preterm (GA<33 weeks) and full term infants when they reached 8 years of life.[4] In accordance with this decline, our analysis did not find an association between birth weight with refractive error in early adolescence (age 12–15 years). This finding could implicate that birth weight has an impact on organ development, but with some delay during childhood refractive error normalizes.

### Astigmatism

In our analysis no association was found between low birth weight and astigmatism. In a Swedish study of infants aged 6 months 18% of former extreme low birth weight infants (≤ 1500g) had an astigmatism of at least 2 diopters.[17] Additionally, Larsson et al. reported that astigmatism declined in a cohort of 198 preterm infants between 6 months and 2.5 years, and afterwards remained stable when analyzed up to 10 years of age.[18] Similarly, other authors reported that the difference between preterm low birth weight infants and full term infants for astigmatism diminishes until 8 to 10 years of age.[4] Our results are in accordance with these studies indicating that astigmatism is not affected by birth weight beyond the age of 10 years.

### Corneal power

In our analysis we found an association between higher corneal power and low birth weight in in early adolescents aged 12 to 15 years. In former preterm and low birth weight infants a smaller corneal radius was observed compared to full term infants from birth up to early childhood.[4, 19, 20] Donzis et al.[21] reported that corneal curvature flattens within 3 months after birth. Other authors hypothesized that a steeper cornea gradually diminishes during infancy.[22, 23] In a previous report, some authors hypothesized that differences between higher intrauterine and lower extrauterine temperature before and after preterm birth may be reasons for a postnatal less flattening of corneal morphology.[24] In accordance with these results, we found an association between low birth weight and higher corneal power, which is inversely related to corneal curvature. This association of corneal power with birth weight was still present after adjustment for several factors, namely sex, age, ethnicity, and NHANES examination cycle.

Other corneal properties, such as central corneal thickness seem to be linked to birth weight as well. Some authors reported thicker central corneal thickness in preterm newborns in comparison to full term newborns.[19, 25] Others reported that the difference of central corneal thickness diminishes in preterm newborns step by step until they reach full term age.[26, 27] Two studies analyzed corneal properties in late infancy using Scheimpflug imaging and reported no difference in central corneal thickness comparing former preterm and low birth weight infants to full term infants.[4, 28] As central corneal thickness is not examined within the National Health and Nutrition Examination Survey, our analysis cannot contribute to this aspect.

### Strengths and limitations

The strength of the NHANES study lies in the standardized population-based study design and the large sample size. Furthermore, the adjustment for different covariates contributes to precise estimates. Several studies detected a high reliability of mother reported birth weight as proxy-reported birth weight parameter.[29–32] Because gestational age was not recorded in this investigation, our analysis could not incorporate if infant’s birth weight was small, appropriate, or large in correlation with gestational age. Furthermore, the fact that cycloplegic measurements were not performed within the comprehensive NHANES examination could also diminish potential differences between study groups. Accommodation may have affected the results for refractive error particularly in the young teenager age. As a consequence hyperopic refractive error might have been underestimated and myopic refractive error overestimated. This has to be considered when interpreting our results. To lower risk of bias, we performed a sensitivity analysis within the myopic study participants and did find similar associations as for the total study cohort. Another limitation of the present study was the small number of participants with very low birth weight (<1500g) which could also diminish possible differences. However, we repeated the analysis of associations with categorized birth weight with an additional category for very low birth weight (<1500 g) and found similar results.

## Conclusion

Overall, this study presents the results of a population-based study of early adolescents aged 12 to 15 years and reports the relationship between birth weight and visual acuity, refractive error, astigmatism, and corneal power. Our results highlight that low birth weight has an impact on corneal configuration even after the first decade of life. Namely corneal power is higher in early adolescents with low birth weight. Due to the inverse correlation between corneal power and corneal curvature this study underlines that low birth weight seems to lead to a less flattening of corneal curvature which persist until early adolescence. Furthermore, the lack of association between birth weight and visual acuity and refractive error could indicate these effects may be limited to infancy and childhood and are not present in early adolescence anymore in a population-based approach.

## Supporting information

S1 Table The association of self-reported birth weight (categorical) with visual acuity, refraction and keratometry in the NHANES 1999–2008, restricted to myopic participants (n = 1553). Legend: Results from the multivariable linear regression models adjusted for age, sex, ethnicity and NHANES examination cycle. Reference was the normal birth weight group (> = 2500 g—< = 4100g). Myopia was defined as spherical equivalent below -0.5 dioptres.
(DOCX)

S2 Table The association of birth weight (continuous) with visual function, refraction and keratometry in the NHANES 1999–2008, restricted to myopic participants (n = 1553). Legend: Results from the multivariable linear regression models adjusted for age, sex, ethnicity and NHANES examination cycle. Myopia was defined as spherical equivalent below -0.5 dioptres.
(DOCX)
