# Correction: Association of birth weight with corneal power in early adolescence: Results from the National Health and Nutrition Examination Survey (NHANES) 1999-2008

**DOI:** 10.1371/journal.pone.0213396

**Published:** 2019-02-28

**Authors:** The PLOS ONE Staff

## Notice of republication

This article was republished on February 21, 2019, to correct for an error in the Data Availability statement introduced during the production process. The publisher apologizes for the error. Please download this article again to view the correct version. The originally published, uncorrected article and the republished, corrected articles are provided here for reference.

## Supporting information

S1 File Originally published, uncorrected article. (PDF)

S2 File Republished corrected article. (PDF)
