# Supplementary material for: Association of birth weight with corneal power in early adolescence: Results from the National Health and Nutrition Examination Survey (NHANES) 1999–2008
Source: PLoS One. 2017 Oct 26;12(10):e0186723. doi: 10.1371/journal.pone.0186723 (PMC5658059; doi:10.1371/journal.pone.0186723)
Supplement: S1 Table — Legend: Results from the multivariable linear regression models adjusted for age, sex, ethnicity and NHANES examination cycle. Reference was the normal birth weight group (> = 2500 g—< = 4100g). Myopia was defined as spherical equivalent below -0.5 dioptres. (DOCX) [file pone.0186723.s001.docx]

**S1 Table. The association of self-reported birth weight (categorical) with visual acuity, refraction and keratometry in the NHANES 1999 - 2008, restricted to myopic participants (n=1553).**

|  | Low birth weight  (<2500 g) |  | High birth weight  (>4100 g) |  |
| --- | --- | --- | --- | --- |
|  | Estimate [95% confidence interval] | P value | Estimate [95% confidence interval] | P value |
| Sphere [dioptres] | 0.19 [-0.21; 0.58] | 0.36 | -0.08 [-0.51; 0.35] | 0.72 |
| Spherical equivalent [dioptres] | 0.19 [-0.18; 0.56] | 0.33 | -0.14 [-0.57; 0.3] | 0.54 |
| Refractive J_0_ | -0.05 [-0.18; 0.08] | 0.49 | 0.05 [-0.05; 0.15] | 0.29 |
| Refractive J_45_ | -0.02 [-0.07; 0.03] | 0.48 | -0.01 [-0.06; 0.03] | 0.56 |

Results from the multivariable linear regression models adjusted for age, sex, ethnicity and NHANES examination cycle. Reference was the normal birth weight group (>=2500 g - <= 4100g). Myopia was defined as spherical equivalent below -0.5 dioptres.
