# Supplementary material for: Association of birth weight with corneal power in early adolescence: Results from the National Health and Nutrition Examination Survey (NHANES) 1999–2008
Source: PLoS One. 2017 Oct 26;12(10):e0186723. doi: 10.1371/journal.pone.0186723 (PMC5658059; doi:10.1371/journal.pone.0186723)
Supplement: S2 Table — Legend: Results from the multivariable linear regression models adjusted for age, sex, ethnicity and NHANES examination cycle. Myopia was defined as spherical equivalent below -0.5 dioptres. (DOCX) [file pone.0186723.s002.docx]

**S2 Table. The association of birth weight (continuous) with visual function, refraction and keratometry in the NHANES 1999 - 2008, restricted to myopic participants (n=1553).**

|  | Estimate per 100g [95 % confidence interval] | P value |
| --- | --- | --- |
| Sphere [dioptres] | -0.01 [-0.03; 0.01] | 0.18 |
| Spherical equivalent [dioptres] | -0.01 [-0.03; 0.01] | 0.24 |
| Refractive J_0_ | 0.01 [0; 0.01] | 0.02 |
| Refractive J_45_ | 0 [0; 0] | 0.38 |

Results from the multivariable linear regression models adjusted for age, sex, ethnicity and NHANES examination cycle. Myopia was defined as spherical equivalent below -0.5 dioptres.
